# First photographic evidence of the Egyptian fruit bat, *Rousettus aegyptiacus* (Pteropodidae) in the King Salman Bin Abdulaziz Royal Nature Reserve, Hail Region, Saudi Arabia

**DOI:** 10.12688/f1000research.169075.5

**Published:** 2026-01-08

**Authors:** Mohammed Bakri, Mohammad Abdulhakeem, Abdulrahman Alasiri, Tariq Aloufi, Noorah Al-Sowayan

**Affiliations:** 1 Department of Biology, College of Science, Qassim University, Buraydah, Saudi Arabia; 2 King Salman Bin Abdulaziz Royal Natural Reserve Development Authority, Al Olaya, Riyadh, 12213, Saudi Arabia

**Keywords:** Chiroptera; Sustainable; Habitat; Distribution; Bats; Hail region; Saudi Arabia

## Abstract

**Background:**

This study aimed to document the occurrence of the Egyptian fruit bat *Rousettus aegyptiacus* in King Salman Bin Abdulaziz Royal Nature Reserve (KSRNR), Hail region, Saudi Arabia, where its presence had not previously been confirmed.

**Methods:**

Field surveys were conducted in the Hail region, and direct observations were documented using photographic and video evidence. The colony was visually counted in the field during a single daytime visit from an estimated distance of 15 meters.

**Results:**

More than 50 individuals, including adults and juveniles, were observed roosting in a rock crevice. The photographic documentation confirms the presence of a colony of *R. aegyptiacus* in this area, extending the known distribution of the species into a previously unrecorded region of Saudi Arabia.

**Conclusions:**

These findings provide the first photographic record of *R. aegyptiacus* in KSRNR and highlight the species’ ecological use of arid rock crevices. The results emphasize the need for targeted surveys and long-term monitoring to better understand the distribution and conservation of this species in desert environments.

## Introduction

The Egyptian fruit bat *Rousettus aegyptiacus* (Geoffroy, 1810), is a frugivorous bat species widely distributed across the Afro-Palearctic region. ^1, 2^ In Saudi Arabia, its confirmed presence has been reported primarily in the southwestern and northwestern parts of the country, including records from Bisha, ^3^ Abha, ^4^ Taima, Al Disah, and Muleh. ^5^ Although some recent studies have extended this range, ^6, 7^ there remains a gap in verified documentation from desert ecosystems in north-central Saudi Arabia.

King Salman Bin Abdulaziz Royal Nature Reserve (KSRNR) spans semi-arid and mountainous landscapes that provide potential roosting and feeding sites for chiropteran species. ^8, 9^ The Aja Mountains in the Hail region, characterized by rocky outcrops and sparse vegetation, may represent suitable bat habitats that are yet to be explored. Bats, particularly those in the family Pteropodidae, play crucial ecological roles and are highly sensitive to environmental changes. ^10^

Based on a comprehensive literature search and available faunal records (e.g., Refs. 6, 7, 11, 12), no previous photographic documentation of this species has been reported within the boundaries of KSRNR.

This study presents the first photographic record of *R. aegyptiacus* in the KSRNR, confirming its presence in Hail and filling a notable gap in the known range. We discuss the potential ecological relevance of this finding and its implications for conservation and biodiversity monitoring in arid zones of Saudi Arabia.

## Methods and materials

### Study area

**Figure 1.**
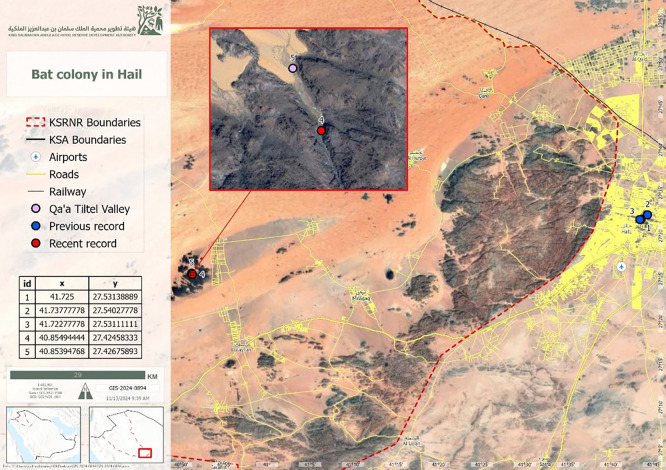
Map of the King Salman Bin Abdulaziz Royal Nature Reserve, Hail Region, showing the study site.

The Hail region is situated on an extensive plateau overlying the Precambrian Arabian Shield, a complex geological formation comprising igneous and metamorphic rock units that exhibit diverse topographic and geomorphic characteristics. ^13^ The study was conducted in the Qa’a Tiltel Valley (27°25′28.5″ N, 40°51′17.8″ E) ( Figure 1), on the western side of Al-Khabbah. The area is characterized by mountainous terrain and semi-desert isolation, and stands out in the landscape as gravelly and sparsely vegetated, dominated by *Haloxylon salicornicom* and *Malva parviflora*, making it an ideal niche for many animal species ( Figure 1).

### Techniques employed

A single daytime field survey was conducted on March 31, 2024, at approximately 9:00 AM by two biodiversity monitors from the KSRNR team. The bat roost was observed from a distance of roughly 15 meters to avoid direct disturbance.

Photographic and video documentation was captured using a Nikon Z5II camera equipped with a 200–600 mm telephoto lens, without using flash.

Individual bats were counted visually during the live observation only, no video footage or still images were used to aid in the count. The count was conducted once and represents an approximate minimum estimate. Age classification into adults and juveniles was based solely on visible features such as body size and fur density, without handling or close-up inspection.

## Results

On March 31, 2024, at 9:00 AM, a roosting colony of over 50 *Rousettus aegyptiacus* was observed in a mountain crevice in the Qa’a Titel area (27°25’28.5”N, 40°51’17.8”E), within the southern part of Hail Region. This site, located in a semi-desert habitat within the boundaries of the King Salman Bin Abdulaziz Royal Nature Reserve, was documented through both photographic and video evidence (see Table 1 for full summary).

**Table 1.** Summary of *Rousettus aegyptiacus* observation in Hail Region.

Observation Date	31 March 2024
Time	9:00 AM
Location	Qa’a Tiltel, Hail Region
Coordinates	27°25′28.5″N, 40°51′17.8″E
Habitat Type	Mountain crevice in semi-desert landscape
Number of Individuals	>50 individuals
Number of Juveniles	±10
Evidence	Photographic and Video

The bats roost in a fissure at the base of a rocky hill within a semi-desert landscape sparsely vegetated with *Haloxylon salicornicum* and *Malva parviflora*. This microhabitat provides shelter and minimal disturbance, which supports bat colonization. These plant species are not considered a food source for the bats but represent part of the natural landscape surrounding the roost.

Photographic and video documentation confirmed identification based on morphology: large body size, strong limbs, short fur with greyish-brown dorsal and ventral coloring, and yellowish markings in some individuals. Juveniles are distinguishable by their lighter color and sparse hair. ^14^ The colony generally exhibited calm behavior with occasional flights in response to disturbances.

Table 1 summarizes the characteristics of the roost site, including location, environmental features, and observed behaviors. The colony included both adults and juveniles. Due to the limitations on physical handling within the protected area, we could not determine the exact number or proportion of each age class. However, based on visible traits-such as smaller body size and lighter fur-we observed that juveniles were present. Most bats were roosting closely together on the cave ceiling, showing minimal activity during the day.

**Figure 2.**
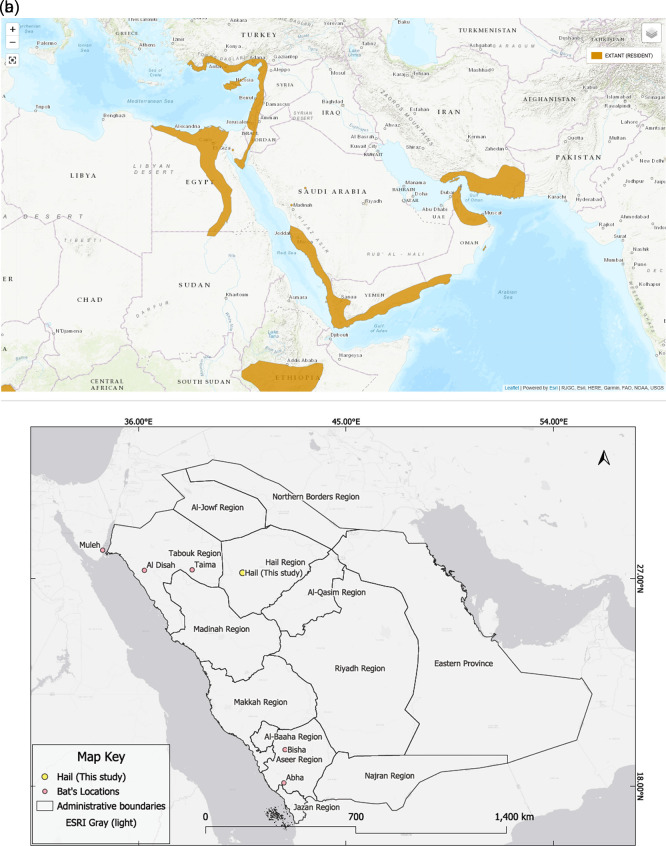
(a) IUCN global distribution range of Rousettus aegyptiacus. (b) Distribution map showing the location of the Egyptian fruit bat ( *Rousettus aegyptiacus*) colony recorded in the King Salman Bin Abdulaziz Royal Nature Reserve (KSRNR), Hail Region, Saudi Arabia. The colony was observed roosting in a rocky hill fissure within a known distributional gap. According to IUCN data, there is no prior record of this species in Saudi Arabia, suggesting this finding fills a distribution gap rather than expanding the known range.

In addition to the new Hail record, six confirmed occurrences of *Rousettus aegyptiacus* have been documented in Saudi Arabia, as shown in Table 2 and Figure 2. These include records from the northern and southern parts of the country based on photographic evidence and published reports. The distribution of *R. aegyptiacus* records across Saudi Arabia is shown in Figure 2.

**Table 2.** Confirmed records of *Rousettus aegyptiacus* in Saudi Arabia with coordinates and references.

Location	Latitude (N)	Longitude (E)	Reference
Taima	27.6	38.6	^6^
Al Disah	28.7	36.3	^6^
Muleh	28.2	35.9	^6^
Hail (New)	27.42	40.85	This study
Bisha	19.98	42.59	^6^^,^^7^
Abha	18.23	42.51	^6^

To provide geographic context for the new record, we added Table 3 to present the estimated distances between the study site in the King Salman Bin Abdulaziz Royal Nature Reserve (Hail Region) and the nearest previously confirmed records of *Rousettus aegyptiacus* in Saudi Arabia. The nearest previous record is from Tayma, approximately 226 km from the study site. This confirms a spatial gap between earlier known locations and the newly observed colony in Hail. This comparison strengthens our statement that this is the first photographic documentation of the species in the Hail Region.

**Table 3.** Distance (in km) between current study site and nearest previously confirmed locations of *Rousettus aegyptiacus*. ^6^

Region	Distance
Taima	226 km
Al Qassim	290 Km
Al Ula	298 km
Tabouk	330 km
Al Madinah Al Munawwarah	338 km

The bat colony was observed in a mountain crevice at 9:00 on March 31, 2024, southern Qa’a Tiltel in the Hail region, containing over 50 bat individuals, which were identified as *R. aegyptiacus* the roost was active inside the cave.

**Figure 3.**
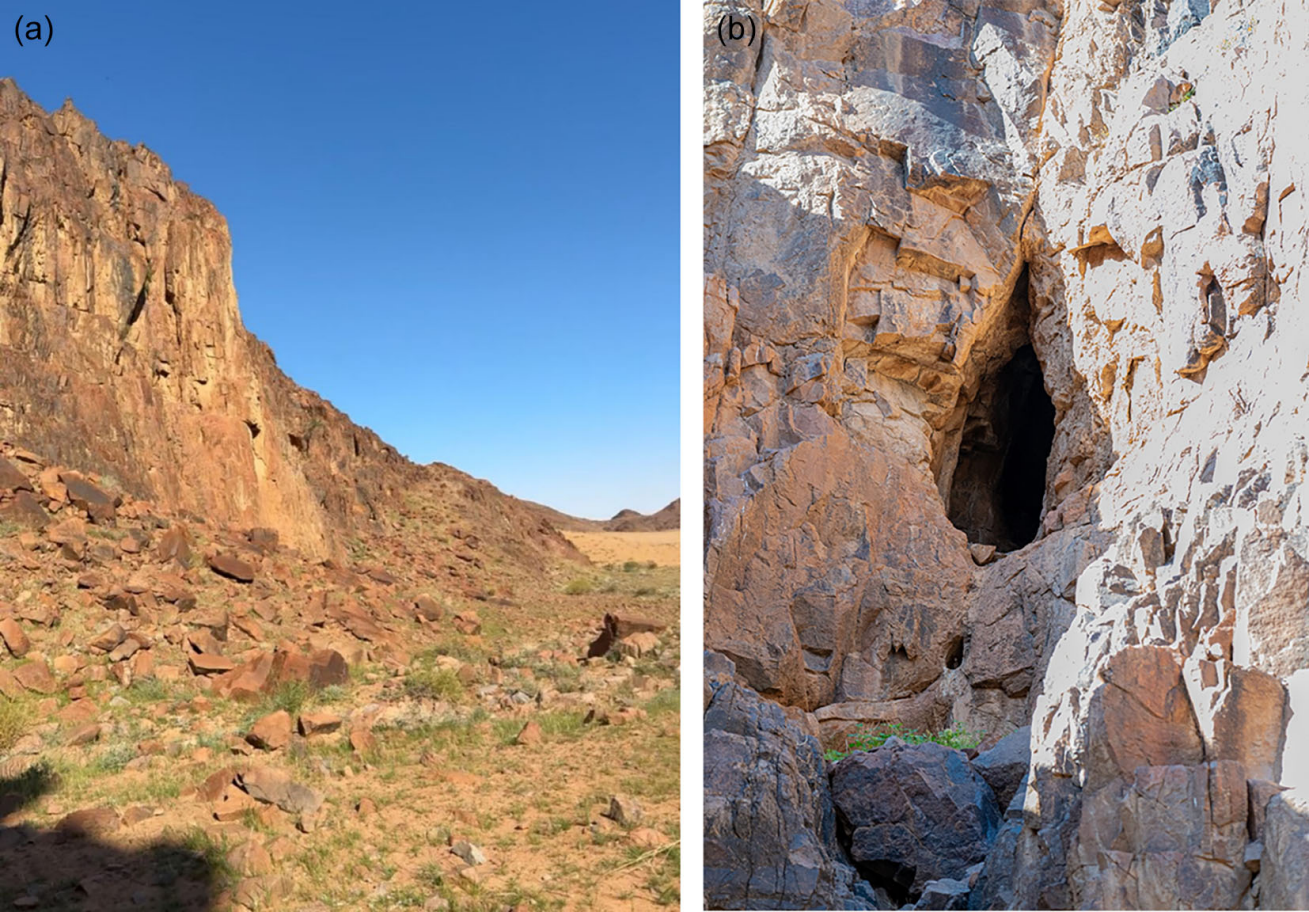
(a) Entrance of the cave (image extracted from Video S2). (b) Detailed view of the cave entrance showing the roosting site and colony of *R. aegyptiacus* recorded in the Hail region (captured by Mohammed Bakri).

**Figure 4.**
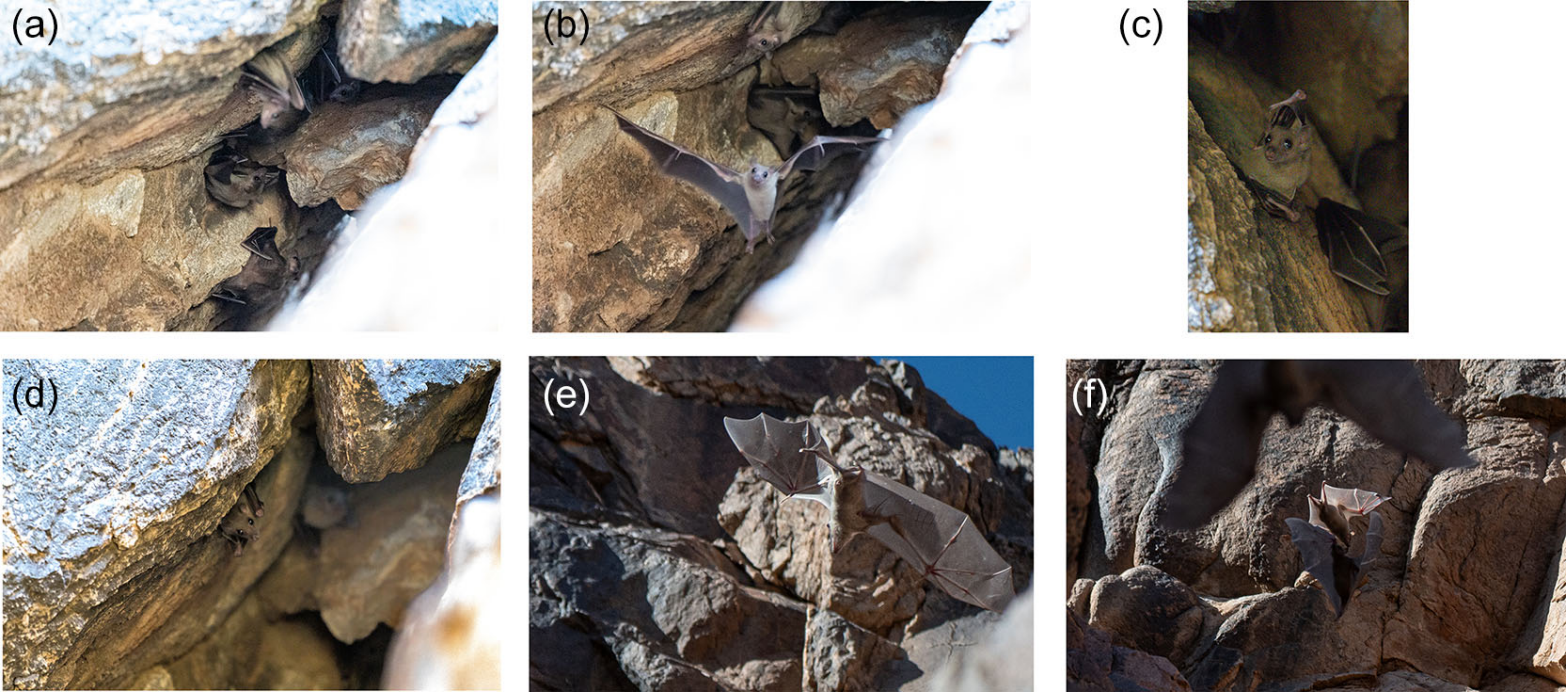
(a–f ) Different individuals of Egyptian fruit bat ( *R. aegyptiacus*) observed on 31 March 2024 (captured by Mohammed Bakri).

The roost was located in a fissure near the bottom of the hill ( Figure 3a and b). The bat colony, consisting of over 50 individuals, was active inside the cave. The presence of this colony in the Hail region, confirmed by photographic documentation, represents a notable faunal record. ^14^ The video recordings and photographs clearly showed large, robust bats with well-developed feet and a strong thumb. The fur was short, and the dorsal and ventral sides appeared uniformly gray or brownish. The belly and throat in some individuals were yellowish. Juveniles were generally gray and more sparsely haired than adults. Photographic evidence of *R. aegyptiacus* in the Hail region is shown in Figure 4a– f and Video 1. *R. aegyptiacus* was active in the cave. This observation confirms the species’ presence in this area.

## Supplementary material

A short video clip (Video S1) showing active individuals of *Rousettus aegyptiacus* within the cave in Qa’a Tiltel is provided as a supplementary material. The footage supports photographic documentation and confirms the roost activity in the area. And Short video (Video S2) of the roost entrance (still image used for Figure 3a).

**Verification of novelty**

To confirm that our finding represents the first photographic record of *Rousettus aegyptiacus* in the Hail region, we conducted an extensive review of available biodiversity documentation. This included:

• Searching the Global Biodiversity Information Facility (GBIF)
• Reviewing national wildlife records from the Saudi Wildlife Authority
• Examining peer-reviewed publications and regional biodiversity surveys

No prior photographic documentation of the species in this region was identified. Therefore, our image constitutes the first visual confirmation of its presence in the King Salman Bin Abdulaziz Royal Nature Reserve.

## Discussion

The confirmed presence of *Rousettus aegyptiacus* in the Hail region particularly within the boundaries of the King Salman Bin Abdulaziz Royal Nature Reserve represents a novel observation. Previous records of this species in Saudi Arabia were largely restricted to the western and southwestern regions (e.g., Makkah and Asir). Its documentation in Hail extends the known distribution range of this frugivorous bat species into the northern part of the Arabian Peninsula, suggesting potential ecological corridors or suitable habitat patches beyond the traditionally recognized range. This finding holds relevance for future conservation planning and highlights the need for broader bat surveys in central and northern Saudi Arabia.

The observed distance between the study site and previously documented locations of *Rousettus aegyptiacus* ranging from 226 to 338 km suggests a broader distribution range than previously recognized. This finding underscores the importance of continued surveys in northern Saudi Arabia, particularly in underexplored regions where ecological data remain scarce.

This report presents the first confirmed photographic evidence of the Egyptian fruit bat *Rousettus aegyptiacus* within the King Salman Bin Abdulaziz Royal Nature Reserve (KSRNR) in the Hail region. Although earlier surveys documented this species in neighboring areas, including Taima, Al Disah, and Muleh, ^5^ and more recently in other parts of Hail, ^7^ these records were either anecdotal or lacked detailed visual verification. This record fills a critical gap by visually confirming the presence of a stable roosting colony within the boundaries of a major conservation area.

This study presents the first photographic evidence of Rousettus aegyptiacus within the boundaries of King Salman Bin Abdulaziz Royal Nature Reserve. Although an earlier non-photographic record was reported from Hail in 1966, ^17^ the current finding confirms the species’ sustained presence and breeding activity inside the protected area, establishing its ecological relevance and conservation priority.

*R. aegyptiacus* is known for its adaptability to diverse environments, from humid forests to arid and semiarid zones. ^1, 5, 11, 15^ Its ability to exploit rocky crevices and caves in dry mountainous areas reflects broader ecological flexibility than previously assumed. Although Harrison and Bates ^12^ and Bergmans ^16^ provided a foundational understanding of the species’ range, recent findings, including those by Benda et al. ^7^ and Al Obaid et al., ^6^ suggested a significant extension of its known habitat, particularly in less-explored northern regions.

The photographic and video documentation in this study adds empirical support to Abu Yaman’s early report by Hail, ^17^ validating historical data and establishing a visual benchmark for future surveys. This visual confirmation strengthens the case when considering the region as a part of the active range of *R. aegyptiacus*.

Further studies are needed to explore other potential roosting sites within the KSRNR, including the unexplored caves and valleys. Regular ecological surveys combined with acoustic monitoring and roost counts are crucial for assessing seasonal movement patterns, reproductive status, and interspecies interactions among local bat communities. Such data are essential to understand the conservation value of desert-protected areas for volant mammals.

## Conclusion

Photographic documentation of *Rousettus aegyptiacus *in the King Salman Bin Abdulaziz Royal Nature Reserve (KSRNR) represents a significant record of the biodiversity of arid zones in Saudi Arabia. This confirmation of a stable colony in the Hail region expands the known geographic range of the species and emphasizes the ecological value of the KSRNR.

This finding supports earlier undocumented sightings in the region and underscores the importance of incorporating visual and photographic verification into biodiversity monitoring. As arid environments face increasing pressure from habitat loss and climate change, conservation of species such as *R. aegyptiacus* requires continuous ecological research and adaptive management strategies.

We recommend initiating systematic surveys across the reserve to identify additional roosting sites and monitor the population health. Integrating local communities and relevant stakeholders into awareness and conservation programmes is critical. Protecting keystone species such as *R. aegyptiacus* contributes to broader ecosystem sustainability goals in Saudi Arabia.

The recommendations are to conduct more thorough surveys to determine the distribution of *R. aegyyeptiacus* throughout various areas of Saudi Arabia in order to obtain higher data rates for this species for better understanding and conservation at the country level and in the entire Arabian Peninsula region. Furthermore, long-term monitoring programs for the population trends and habitat selection of this species have been conducted. Working with local communities and key stakeholders is critical for creating awareness, which helps them to engage in conservation initiatives. Finally, initiatives geared toward the conservation of the Egyptian fruit bat also serve broader purposes in supporting biodiversity and maintaining desert ecosystem sustainability within Saudi territory.

## Ethics statement

This study was purely observational and did not involve capture, handling, disturbance, or experimental manipulation of animals. Permission to conduct observations within the King Salman Bin Abdulaziz Royal Nature Reserve was obtained from the Reserve authorities. Therefore, separate ethical approval was not required. The bats were observed and photographed from a distance in their natural habitat without any interference.

## Data Availability

Zenodo: First photographic evidence of the Egyptian fruit bat in Saudi Arabia. https://doi.org/10.5281/zenodo.17037131 ^18^ This project contains the following underlying data:
•
Figure files (JPEG): Original images of Figures 1–4. • Video files (MP4): Video S1 (colony activity), Video S2 (crevice entrance, still image used as Figure 3a). •
Table files (DOCX): Observation records (Tables 1 and 2). Zenodo: Supplementary materials. https://doi.org/10.5281/zenodo.17037131 ^18^ This project contains the following extended data:
• Supplementary Table S1. Details of *R. aegyptiacus* observations in the King Salman Bin Abdulaziz Royal Nature Reserve, Hail Region, Saudi Arabia. • Supplementary Table S2. Confirmed records across Saudi Arabia with coordinates and references. Data are available under the terms of the Creative Commons Attribution 4.0 International license (CC-BY 4.0).
