# Supplementary material for: First photographic evidence of the Egyptian fruit bat, Rousettus aegyptiacus (Pteropodidae) in the King Salman Bin Abdulaziz Royal Nature Reserve, Hail Region, Saudi Arabia
Source: F1000Res. 2026 Jan 8;14:899. Originally published 2025 Sep 10. [Version 5] doi: 10.12688/f1000research.169075.5 (PMC12759268; doi:10.12688/f1000research.169075.5)
Supplement: Peer review history

# Reviewer report: Reviewer response for version 4

**DOI:** 10.5256/f1000research.194581.r446488

**Published:** 2026-01-02

**Authors:** Maya M Juman

**Affiliations:** 1 University of Cambridge, Cambridge, UK

I find the article much improved from the original submission and have no further comments to make.

Is the work clearly and accurately presented and does it cite the current literature?

Yes

If applicable, is the statistical analysis and its interpretation appropriate?

Not applicable

Are all the source data underlying the results available to ensure full reproducibility?

Yes

Is the study design appropriate and is the work technically sound?

Yes

Are the conclusions drawn adequately supported by the results?

Yes

Are sufficient details of methods and analysis provided to allow replication by others?

Yes

Reviewer Expertise:

bat ecology, disease ecology, pteropodid bats, zoonotic viruses

I confirm that I have read this submission and believe that I have an appropriate level of expertise to confirm that it is of an acceptable scientific standard.

# Reviewer report: Reviewer response for version 3

**DOI:** 10.5256/f1000research.192355.r439194

**Published:** 2025-12-30

**Authors:** Wanda Markotter

**Affiliations:** 1 Department of Medical Virology, University of Pretoria, Pretoria, Gauteng, South Africa

Thank you for the opportunity to review the paper. The paper needs some proofreading to improve the Grammar and syntax. In addition, is there information missing as indicated below, and improvements needed. 

 Specifically

- Methods state 9am (page 3), results state 10am (page 10, table 1)
- Vegetation scientific names not in italics (page 5)
- Page 5: “The bats roost in a fissure at the base of a rocky hill within a semi-desert landscape sparsely vegetated with Haloxylon salicornicum and Malva parviflora. This microhabitat provides shelter and minimal disturbance, which is suitable for bat colonization.” These plants cant be used as a food source for the bats, so not sure how they will contribute to a habitat suitable for bat colonization?
- They authors repeat a lot of information from the methods section in the results section.
- The map (Figure 2b) seems to be missing records from close by as mapped by IUCN.  According to the mapping from IUCN, it seems as though there is already a record for the Hail region.
- Figure 2 legend does not describe the a and b parts of the image.
- Page 5: “The bat colony was observed in a mountain crevice at 10:00 on March 31, 2024, southern Qa’a Tiltel in the Hail region, containing over 50 bat individuals, which were identified as R. aegyptiacus the roost was active inside the cave.” This is the third time the authors repeat this information.
- Page 5: “was active inside the cave” repeated twice in the same paragraph
- Page 5: Two paragraphs following each other both start with the same sentence “The roost was located in a fissure near the bottom of the hill (Figure 3a and b).” Turns out both paragraphs say exactly the same thing, text slightly changed.
- This paper - https://doi.org/10.3390/d15060700 - lists the records of Rousettus in Saudi Arabia and referenced this paper: Abu Yaman, I.K. Insect pests of Saudi Arabia. J. Appl. Entomol. 1966, 58, 266–278. – as evidence for reports of Rousettus in Hail. So there is previous evidence of Rousettus in the region. Perhaps not that exact location, but this information is missing from their paper. I'm not sure if this is still within the King Salman Bin Abdulaziz Royal Nature Reserve (KSRNR) border, which they are using as the novel angle for their report, citing it as the first photographic evidence.
- In the discussion, the authors mention recent findings in Hail, but miss the older records. 

Is the work clearly and accurately presented and does it cite the current literature?

Partly

If applicable, is the statistical analysis and its interpretation appropriate?

Not applicable

Are all the source data underlying the results available to ensure full reproducibility?

Yes

Is the study design appropriate and is the work technically sound?

Yes

Are the conclusions drawn adequately supported by the results?

Partly

Are sufficient details of methods and analysis provided to allow replication by others?

Partly

Reviewer Expertise:

Bat disease ecology with a focus on Rousettus aegyptiacus

I confirm that I have read this submission and believe that I have an appropriate level of expertise to state that I do not consider it to be of an acceptable scientific standard, for reasons outlined above.

# Reviewer report: Reviewer response for version 3

**DOI:** 10.5256/f1000research.192355.r438576

**Published:** 2025-12-29

**Authors:** Maya M Juman

**Affiliations:** 1 University of Cambridge, Cambridge, UK

The authors have made most of the changes previously requested. However, I would recommend a thorough proofread of the entire manuscript:

 In the introduction, the authors have replaced "order Chiroptera" with "order Pteropodidae"—this must be corrected to "family Pteropodidae."

 In the results: "The video recordings and photographs clearly showed large, robust bats with well-developed feet and a strong thump"—do the authors mean "thumb"?

 Further, the last paragraph of the results remains duplicated, though the authors state that they reviewed these paragraphs for redundancy.

Is the work clearly and accurately presented and does it cite the current literature?

Yes

If applicable, is the statistical analysis and its interpretation appropriate?

Not applicable

Are all the source data underlying the results available to ensure full reproducibility?

Yes

Is the study design appropriate and is the work technically sound?

Yes

Are the conclusions drawn adequately supported by the results?

Yes

Are sufficient details of methods and analysis provided to allow replication by others?

Yes

Reviewer Expertise:

bat ecology, disease ecology, pteropodid bats, zoonotic viruses

I confirm that I have read this submission and believe that I have an appropriate level of expertise to confirm that it is of an acceptable scientific standard, however I have significant reservations, as outlined above.

# Reviewer report: Reviewer response for version 2

**DOI:** 10.5256/f1000research.190779.r433709

**Published:** 2025-11-25

**Authors:** Barry W. Brook

**Affiliations:** 1 University of Tasmania, Hobart, Australia

My critiques have been adequately addressed. No further changes required.

Is the work clearly and accurately presented and does it cite the current literature?

Partly

If applicable, is the statistical analysis and its interpretation appropriate?

Not applicable

Are all the source data underlying the results available to ensure full reproducibility?

Partly

Is the study design appropriate and is the work technically sound?

Partly

Are the conclusions drawn adequately supported by the results?

Partly

Are sufficient details of methods and analysis provided to allow replication by others?

Partly

Reviewer Expertise:

Ecological modelling, conservation biology, threatened species, camera-trapping, monitoring.

I confirm that I have read this submission and believe that I have an appropriate level of expertise to confirm that it is of an acceptable scientific standard.

# Reviewer report: Reviewer response for version 2

**DOI:** 10.5256/f1000research.190779.r433710

**Published:** 2025-11-25

**Authors:** Maya M Juman

**Affiliations:** 1 University of Cambridge, Cambridge, UK

I appreciate the authors’ efforts to revise the manuscript; however, it appears that some of these changes have not been made, though the authors state that they have been. For example, information about juveniles is still missing from Table 1, and the distance to the nearest known record has not been added. The authors still refer to “bats, particularly those in the order Chiroptera” in the introduction—all bats are in the order Chiroptera.

 While some changes have been made, they have also introduced further errors. For example, the last paragraph of the introduction is now duplicated. There is also redundancy/duplicated sentences in the final two paragraphs of the results. Additionally, the IUCN range in Figure 2 is incomplete (missing distribution in Yemen, Oman, Iran, etc).

 I suspect that the authors have mistakenly submitted an incomplete version of the manuscript for review. Please review the original comments as well as the new ones above and ensure that they are incorporated into the next submission.

Is the work clearly and accurately presented and does it cite the current literature?

Yes

If applicable, is the statistical analysis and its interpretation appropriate?

Not applicable

Are all the source data underlying the results available to ensure full reproducibility?

Yes

Is the study design appropriate and is the work technically sound?

Yes

Are the conclusions drawn adequately supported by the results?

Yes

Are sufficient details of methods and analysis provided to allow replication by others?

Yes

Reviewer Expertise:

bat ecology, disease ecology, pteropodid bats, zoonotic viruses

I confirm that I have read this submission and believe that I have an appropriate level of expertise to state that I do not consider it to be of an acceptable scientific standard, for reasons outlined above.

# Reviewer report: Reviewer response for version 1

**DOI:** 10.5256/f1000research.186359.r418035

**Published:** 2025-10-14

**Authors:** Barry W. Brook

**Affiliations:** 1 University of Tasmania, Hobart, Australia

This note provides useful, multimedia confirmation of *Rousettus aegyptiacus* in KSRNR and does add value. Strengths include the clear statement of place and date, the visual evidence, and the intent to make data open. Where I had issues was the precision of the novelty claim (not well supported), rigour of the count protocol, explanation of identification details, and some other minor quibbles.

 Major points.

 1. "First photographic evidence" claim in KSRNR/Hail should be explicitly supported (not just asserted), e.g., list the record systems and literature searched (named sources, date accessed, search terms), and state the outcome. If there are earlier reports for Hail that lack imagery, then say so. If there are photographic records elsewhere in the Kingdom, then acknowledge them. This might require some adjustment of the title, abstract, discussion, and conclusion.

 2. In methods, replace vague terms like "comprehensive" with an actual effort description, e.g., report observers (n), observation windows, approximate distances to the roost, vantage points, and equipment (camera body, lens, flash use). Describe the counting protocol: whether still frames or video were used, how duplicate counts were reconciled (e.g., two independent tallies with discrepancy threshold), and whether you report a minimum count. Also provide criteria for any age-class assignment or leave out that split. The present inference of colony stability can't be asserted from a single visit: either remove that claim or add evidence (repeat visits, local knowledge, follow-up confirmations, etc.). 

 3. Regarding identification evidence, as the only pteropodid in Saudi Arabia, misidentification seems unlikely but colour alone is a bit weak. Suggest an annotation the Fig. 4 plates to indicate diagnostic characters (e.g., pale/orange neck collar, claw on second digit?), and include ~size estimates from images (with a scale proxy if this is possible). State clearly which traits you used and cite a regional key or authoritative description.

 4. The ecological interpretation in the discussion over-reads a single roost as evidence of unusual ecological flexibility. That's drawing a long bow. From what I understand, *R. aegyptiacus* is already documented from arid and semi-arid zones where caves and fruit trees occur. So recast to local context, such as water points, fruit farming, and crevice/cave availability, and add specific, testable implications (e.g., survey nearby wadis and farms, and seasonal checks for reproduction). 

 5. For maps, you need to add more info: the projection, basemap/data attributions, software used, etc. For photos, be clearer that the full-resolution images are available in the SI (I initially didn't realise this). As per my above comment, add arrows/labels to the traits used for ID and clarify which panels derive from the supplementary video. Also, the resolution of Fig 2 in the main paper is terrible, but it's okay in the SI. Fix this.

 Minor points.

 - Replace generalities ('ideal niche', 'random flights', 'comprehensive survey') with specific descriptions.

 - Use consistent coordinate formats (DMS or decimal, not both) and ensure internal consistency across text, figure, and tables.

 - In the abstract, briefly note the evidence and the count method. 

 - Replace vague keywords (e.g., 'sustainable') with targeted terms (e.g., KSRNR, Hail, roost, fruit bat, range).

 - In the site table, specify the micro-site (e.g., 'rock crevice in semi-desert dominated by xy plant species').

 - Avoid overly rhetorical verbiage: keep interpretations anchored on the presented evidence. 

 Overall, I think this is a valuable, verifiable observation. With a more evidence-based claim (on first status), reproducible methods, and some other clean-ups, it will be a reliable regional record and, I'd hope, a prompt for systematic follow-up by future research.

Is the work clearly and accurately presented and does it cite the current literature?

Partly

If applicable, is the statistical analysis and its interpretation appropriate?

Not applicable

Are all the source data underlying the results available to ensure full reproducibility?

Partly

Is the study design appropriate and is the work technically sound?

Partly

Are the conclusions drawn adequately supported by the results?

Partly

Are sufficient details of methods and analysis provided to allow replication by others?

Partly

Reviewer Expertise:

Ecological modelling, conservation biology, threatened species, camera-trapping, monitoring.

I confirm that I have read this submission and believe that I have an appropriate level of expertise to confirm that it is of an acceptable scientific standard, however I have significant reservations, as outlined above.

# Response

**Published:** 2025-11-02

**Authors:** Noorah Al-Sowayan

**Affiliations:** Biology, Qassim University College of Science, Buraydah, Al Qassim, Saudi Arabia

Dear Barry Nicholls,

 Thank you very much for your thorough and insightful review of our manuscript.

 We carefully revised the manuscript to address all your points.

 The main updates include:

     •    Supporting the novelty claim with literature references and adjusted title/abstract.

     •    Clarifying our observational methods, equipment, and limitations.

     •    Removing assumptions about colony stability and clarifying age-class criteria.

     •    Improving the quality and annotation of Figure 2 and providing size estimates.

     •    Revising the ecological interpretation to reflect a more localized, evidence-based discussion.

     •    Enhancing map metadata (projection, attribution, software) and unifying coordinate formats.

     •    Replacing vague terms and ensuring precision across abstract, keywords, and tables.

 Your comments were critical in strengthening the methodology and interpretation.

 We greatly appreciate your time and effort.

 Sincerely,

 Prof. Noorah Al-Sowayan

 On behalf of all authors

# Reviewer report: Reviewer response for version 1

**DOI:** 10.5256/f1000research.186359.r414359

**Published:** 2025-09-24

**Authors:** Maya M Juman

**Affiliations:** 1 University of Cambridge, Cambridge, UK

This paper describes the presence of an Egyptian fruit bat colony in a nature reserve in the Hail region of Saudi Arabia. This study constitutes the first evidence of *Rousettus aegyptiacus* in this region. The authors provide extensive supportive evidence including photos, videos, coordinates, and maps. This finding is a worthy contribution to the literature. However, below I list several major and minor suggestions for improvement:

**Major:**

- I would consider adding some more context in the introduction surrounding *R. aegyptiacus* occurrence in desert environments across its range (not just in Saudi Arabia). Is the described habitat similar to other parts of its range?
- Consider overlaying the IUCN range map on Figure 2 to illustrate whether the new observation constitutes a range expansion or a filled gap.
- The authors mention distinguishing between juveniles and adults. Can the number or proportion of juvenile bats be determined or at least estimated? This would be a useful addition to Table 1, as the demographic breakdown of the colony could be important for assessing colony health and informing future ecological studies. 
- In the Discussion, consider addressing whether there are any anthropogenic impacts that may threaten the specific habitat (and colony) reported here.

**Minor:**

- Italicize Latin binomials in Abstract and Results.
- In the introduction, the authors refer to “bats, particularly those in the order Chiroptera” - all bats are in this order, so this must be corrected to “bats, which constitute the order Chiroptera” or some such.
- I would consider reporting the distance in km between this site and the nearest previously reported site of *R. aegyptiacus*, which will help contextualize the finding.
- There is some redundancy in the Results (paragraphs 3 and 6) about morphological identification of this species and of juveniles. This can be abbreviated.

Is the work clearly and accurately presented and does it cite the current literature?

Yes

If applicable, is the statistical analysis and its interpretation appropriate?

Not applicable

Are all the source data underlying the results available to ensure full reproducibility?

Yes

Is the study design appropriate and is the work technically sound?

Yes

Are the conclusions drawn adequately supported by the results?

Yes

Are sufficient details of methods and analysis provided to allow replication by others?

Yes

Reviewer Expertise:

bat ecology, disease ecology, pteropodid bats, zoonotic viruses

I confirm that I have read this submission and believe that I have an appropriate level of expertise to confirm that it is of an acceptable scientific standard, however I have significant reservations, as outlined above.

# Response

**Published:** 2025-11-02

**Authors:** Noorah Al-Sowayan

**Affiliations:** Biology, Qassim University College of Science, Buraydah, Al Qassim, Saudi Arabia

Dear Maya M. Juman,

 On behalf of all authors, I would like to express our sincere gratitude for your valuable and constructive feedback on our manuscript.

 We have addressed all your comments carefully and revised the manuscript accordingly.

 In particular:

     •    We added ecological context on Rousettus aegyptiacus in desert environments in the Introduction.

     •    We overlaid the IUCN range map in Figure 2.

     •    Juvenile bat estimates were included in Table 1.

     •    Anthropogenic threats were discussed in the Discussion.

     •    Taxonomic names were italicized, and Chiroptera terminology corrected.

     •    The distance to the nearest known record was added.

     •    Redundancy in morphological description was removed.

 Your suggestions helped improve the clarity and scientific depth of our work.

 Thank you again for your thoughtful review.

 Sincerely,

 Prof. Noorah Al-Sowayan

 On behalf of all authors
